# Description of the hemipenial morphology of *Tupinambis quadrilineatus* Manzani and Abe, 1997 (Squamata, Teiidae) and new records from Piauí, Brazil

**DOI:** 10.3897/zookeys.361.5738

**Published:** 2013-12-12

**Authors:** Marcélia Basto da Silva, Geraldo Rodrigues de Lima-Filho, Áurea Aguiar Cronemberger, Leonardo Sousa Carvalho, Paulo Roberto Manzani, Jânia Brito Vieira

**Affiliations:** 1 Museu Paraense Emilio Goeldi, Departamento de Zoologia, Av. Perimetral, 1901, Bairro Terra Firme, CEP 66077-530, Belém, PA, Brazil; 2 Universidade Federal do Piauí, Campus Amílcar Ferreira Sobral, BR 343, KM 3.5, Bairro Meladão, s/nº, CEP 64800-000, Floriano, PI, Brazil; 3 Universidade Estadual de Campinas, Instituto de Biologia, Departamento de Biologia Animal, Rua Monteiro Lobato, 255, Distrito de Barão Geraldo, CEP 13083-862, Campinas, SP, Brazil; 4 Universidade Federal do Piauí, Campus Ministro Reis Veloso, Avenida São Sebastião 2819. CEP. 64202-220, Parnaíba, PI, Brazil

**Keywords:** Hemipenis, systematics, Tupinambinae, *Tupinambis*, new records

## Abstract

Few data are available on the morphology of the hemipenis of teiid lizards, especially those of the recently-defined genus *Tupinambis*, a widely-distributed group of large-bodied lizards. This study provides an illustrated description of the hemipenis of *Tupinambis quadrilineatus*, which is similar to that of other representatives of the Tupinambinae subfamily. New records of the species from the state of Piauí, in northeastern Brazil, are also presented.

## Introduction

The genus *Tupinambis* Daudin (Teiidae) comprises a group of large (maximum SVL of 400 mm) Neotropical lizards, which are distinguished from all other teiids by the combination of smooth dorsal scales, a single loreal, a gap in the granular scales separating the femoral from the abdominal pores, and a cylindrical tail with complete annuli alternating with annuli divided on the dorsal and lateral sides (Harvey et al. 2012). In a recent review of the Teiidae, Harvey et al. (2012) resurrected the genus *Salvator* Duméril and Bibron to refer to the species of the “southern clade” (*sensu* Fitzgerald et al. 1999) previously included in *Tupinambis*. These species are now known as *Salvator merianae* (Duméril & Bibron, 1839), *Salvator rufescens* (Günther, 1871) and *Salvator duseni* (Lönnberg, 1896). According to this scheme, the genus *Tupinambis* currently includes only the four species of the northern or “Amazonian” clade (*sensu* Fitzgerald et al. 1999) – *Tupinambis longilineus* Avila-Pires, 1995, *Tupinambis palustris* Manzani & Abe, 2002, *Tupinambis quadrilineatus* Manzani & Abe, 1997, and *Tupinambis teguixin* (Linnaeus, 1758). The genus *Tupinambis* is found in Colombia, Venezuela, Trinidad and Tobago, the Guyanas, the Amazon basin, and the savannas of Bolivia and Brazil (Harvey et al. 2012). Despite the conspicuous size of these lizards, zoogeographic data are sketchy, and new localities have been recorded recently for some species, such as *Tupinambis longilineus* (Lima and Pimenta 2008, Costa et al. 2008) and *Tupinambis quadrilineatus* (Ferreira et al. 2009, Silveira 2009).

*Tupinambis quadrilineatus* is endemic to the Cerrado savannas of central Brazil. The species was described in 1997, based on four specimens from Goiás, Mato Grosso, and Tocantins (Manzani and Abe 1997, Silveira 2009). Almost simultaneously, Colli et al. (1998) described the same form under the junior-synonym *Tupinambis cerradensis*. A number of other specimens were collected subsequently in the Brazilian states of Goiás, Minas Gerais, Mato Grosso, Maranhão, Tocantins, Piauí, Pará and the Distrito Federal (Barreto et al. 2007, Ferreira et al. 2009, Silveira 2009, Dal Vechio et al. 2013). The geographic range of the species is extended further in the present study.

The hemipenis of *Tupinambis quadrilineatus* is also described here for the first time. The hemipenial morphology of teiid lizards is poorly known (Harvey et al. 2012). Cope (1896) analyzed the hemipenis of the genera *Dracaena*, *Tupinambis*, *Ameiva*, and *Cnemidophorus* and concluded that the morphology of these typical teiid species consist of numerous delicate, imbricate, transverse laminae, which are closely attached to one another. Dowling and Duellman (1978, figure 83.2) published an illustration of the sulcate surface of the hemipenis of a species referred to as *Tupinambis nigropunctatus* Spix, 1825, however they did not provide a museum number, nor did they describe the organ. Presently, *Tupinambis nigropunctatus* is considered as a synonym of *Tupinambis teguixin* (Linnaeus, 1758), and its drawing exihibited a slightly bilobed and relatively long hemipenis, with distal laminae. In addition, the hemipenial morphology of 13 teiid species was described by Böhme (1988), but the author did not examine nor describe the hemipenis of *Tupinambis*.

Besides, Harvey et al. (2012) reviewed the taxonomy and phylogeny of the teiids and included descriptions of the hemipenes of a number of species of the subfamily Tupinambinae, including *Crocodilurus amazonicus* Spix, 1825 and *Salvator merianae*. This study shows that the hemipenis in the Tupinambinae can be characterized as an organ with transverse laminae, a pair of apical awns, and catchment folds. Awns are usually prominent subcylindrical structures, rounded at their distal ends, located at the apex of the lobes. The most elaborate sulcate catchment fold can be observed in *Crocodilurus* and *Salvator*, in which the portion of the fold closest to the sulcus projects outward as a prominent triangular flap. A summary of the hemipenial characters for the Teiidae subfamilies presented by Harvey et al. (2012) is shown in Table 1. The hemipenial morphology of *Tupinambis* nevertheless remains unknown, and the present paper provides a first detailed description of the organ in this genus.

**Table 1.** Hemipenial characters of teiid lizards of three subfamilies of Teiidae (Harvey et al. 2012).

Subfamilies	Proximal laminae	Distal laminae	Discontinuous laminae	Awns	Apical sulcate<br/> Structures	Apical asulcate structures
Callopistinae	6	19	Present	Absent	Large Flat<br/> Expansions	Absent
Teiinae	0–50	5–24	Absent (in most species)	Present	Catchment Fold/ Papillate/ Subtriangular or Rounded Lobes	Rounded Lobes/<br/> High semicircular or Straight Ridges (Flap)/ Subtriangular<br/> Flaps/ Papillate/ Rounded Lobes
Tupinambinae	27–40	44–71	Absent	Absent/Styloid	Catchment Folds with Triangular Flaps	Rounded Lobes

## Methods

Specimens were collected from a locality in the Cerrado savanna of the state of Maranhão and different phytophysiognomies in Piauí. The material examined is deposited in the herpetological collections of the Coleção de História Natural of the Universidade Federal do Piauí, Floriano, Piauí (CHNUFPI, curator: L. S. Carvalho) and the Museu Paraense Emílio Goeldi, Belém, Pará (MPEG, curator: A. L. C. Prudente). Museum abbreviations follow Levington et al. (1980). Scale counts, body measurements, and color pattern are based on the schemes of Manzani and Abe (1997) and Colli et al. (1998). The sex of the specimens was determined by the presence or absence of a hemipenis verified through an incision at the base of the tail. Hemipenis terminology follows Savage (1997), Myers et al. (1993) and Harvey et al. (2012), and the specimens were prepared as in Pesantes (1994), Manzani and Abe (1988) and Zaher and Prudente (2003).

## Taxonomy

### Tupinambis quadrilineatus

Manzani & Abe, 1997

http://species-id.net/wiki/Tupinambis_quadrilineatus

Tupinambis quadrilineatus Manzani & Abe, 1997: 2 (adult male holotype deposited in the Museu de Zoologia of the Universidade Estadual de Campinas, ZUEC 1963, type-locality: Fazenda Bandeirantes, Municipalty of Baliza, Goiás, Brazil (16°13'S, 51°25'W, SAD69), not examined).
Tupinambis cerradensis Colli, Péres & Cunha, 1998: 479 (adult male holotype deposited in the Coleção de Herpetologia of the Universidade de Brasília, CHUNB 00468, type-locality: Rosário Oeste, Mato Grosso, Brazil (14°50'S, 56°25'W, SAD69), not examined).
Tupinambis quadrilineatus ; Taylor 2003: 44, Langstroth 2005: 106, Silva Jr. et al. 2005: 81, Vitt et al. 2005: 8, Werneck and Colli 2006: 1987, Guimarães et al. 2007: 353, Recoder and Nogueira 2007: 270, Silveira 2009: 442, Ferreira et al. 2009: 355, Moreira et al. 2009: 187, Recoder et al. 2011: 275.

#### Diagnosis.

*Tupinambis quadrilineatus* differs (see Table 2 for scale counts and measurements) from the other species of the genus in the presence of 11–18 femoral pores (15–18 in *Tupinambis teguixin*, 20–22 in *Tupinambis longilineus*, 18–26 in *Tupinambis palustris*), 94–118 scales around the mid-body (94–124 in *Tupinambis teguixin*, 90–98 in *Tupinambis longilineus*, 112–119 in *Tupinambis palustris*), 113–138 dorsal scales (102–126 in *Tupinambis teguixin*, 110–121 in *Tupinambis longilineus*, 111–122 in *Tupinambis palustris*) and the coloration. In *Tupinambis quadrilineatus*, the upper lateral stripe is well-defined along the flanks, whereas in other species, it is indistinct or absent (Avila-Pires 1995, Colli et al. 1998, Manzani and Abe 2002, Harvey et al. 2012).

**Table 2.** Scale counts of the specimens of *Tupinambis quadrilineatus* analyzed in the present study and the known range of values for the species, according to Manzani and Abe (1997) and Colli et al. (1998).

Character	CHNUFPI 0036	CHNUFPI 0037	CHNUFPI 0038	MPEG 16817	MPEG 16845	MPEG 30139	MPEG 30140***	MPEG 30141	Known range of values
Sex	Male	Male	Male	Female	Female	Male	Immature male	Male	
Femoral pores*	11	10	12	10	10	11	11	11	11–18
Pre-cloacal pores*	10	8	10	8	9	8	8	8	5–11
Dorsal scales	127	118	119	115	116	109	111**	117	113–138
Scales around midbody	116	105	120**	112	116	90**	103	98	94–118
Ventral scales in a transverse row	24	24	25	25	26	23	24	25	20–28
Lamellae under fourth finger	15	13	15	14	15	14	15	14	12–17
Lamellae under fourth toe	29	30	30	33	34**	27	32	27	26–33
Loreal scale	1	1	1	1	1	1	1	1	1
Supralabial scales*	17**	15	17**	16	16	-	-	-	13–16
Infralabial scales*	14	14	16	14	14	-	-	14	13-17
Snout-vent length (mm)	260	260	227	245	235	260	135	255	88-270
Body width (mm)	49.56	54.48	48.36	59.98	51.27	58.66	-	55.98	17.92-61.86
Body height (mm)	36.58	37.20	30.46	42.09	36.03	44.59	-	40.66	13.95-51.98
Head length (mm)	55.66	55.92	51.29	52.05	52,80	54.32	-	52.39	24.10-62.04
Head width (mm)	42.73	44.98	34.98	41.61	33.91	39.44	-	40.22	15.16-44.28
Head height (mm)	30.20	28.37	28.26	35.47	30.35	36.23	-	29.69	11.60-38.40

* Total number on both sides.

** Exceeds maximum value recorded in previous studies.

***This specimen has a damaged head and part of the body, which prevented the withdraw of the remaining measures.

#### Hemipenial morphology.

**Figure 1.**
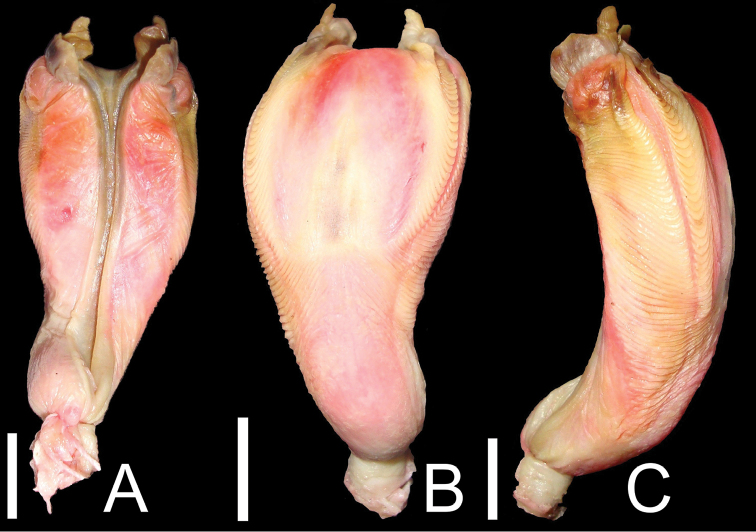
Right hemipenis of *Tupinambis quadrilineatus* (CHNUFPI 0036). **A** sulcate surface **B** asulcate surface **C** lateral region. Scale bar = 1 cm.

The hemipenis of three specimens of *Tupinambis quadrilineatus* (CHNUFPI 0036, CHNUFPI 0038 and MPEG 30139) were prepared for analysis. The organ is relatively long, robust and slightly bilobed, with a total length of 5.0 cm and a width of 2.0 cm in the distal portion of the body (Figure 1). When inverted, the organ extends as far as the fifteenth subcaudal scale. Sulcus spermaticus bifurcated, deep and centripetal. Edge of the sulcus spermaticus pronounced along its entire length. The point of bifurcation of the lobes extends inwardly towards the central region of the styloid process. A pair of short and prominent lobes (about 16% of the total size of the organ) in the form of styloid process are present on either side of the sulcate and asulcate surface, with a pair of catchment folds (extensions of the lips of the sulcus, in the form of prominent sulcal flaps, with rounded edges) coating the styloid process. The region between the lobes is smooth on both the sulcate and asulcate surfaces. Naked sulcate and asulcate expansion pleat. Between 35 and 38 distal laminae (mean = 36 ± 1, n = 3), arranged in a transverse row on each side, extending from just below the apical folds to the base of the lobes. A lateral sulcus separates the distal laminae from the sulcate and asulcate surfaces. Fifteen to 17 proximal laminae (mean = 16 ±1, n = 3). Basal region smooth on the sulcate surface, and wrinkled on the asulcate surface. Discontinuous laminae and basal papillae absent.

The hemipenial morphology of *Tupinambis quadrilineatus* is similar to that of other Tupinambinae in the ornamentation of the body, which are bilobed and have lamelae (Cope 1896, Dowling and Duellman 1978, Harvey et al. 2012). As in *Salvator merianae*, *Tupinambis teguixin* and *Crocodilurus amazonicus* (Dowling and Duellman 1978, Harvey et al. 2012), the hemipenis of *Tupinambis quadrilineatus* lacks the discontinuous distal laminae seen in *Ameiva ameiva* and *Ameivula ocellifera*. However, *Salvator merianae*, formerly considered to be a member of the genus *Tupinambis*, has a relatively long hemipenis, which lacks the lateral and medial expansion pleats and has more laminae (distal laminae: 56–71 and proximal laminae: 33–40) than other teiids (Harvey et al. 2012). See Table 1 for the differences in the hemipenial morphology of three subfamilies of Teiidae (Harvey et al. 2012). The morphology and ornamentation of the hemipenis play an important role in the diagnosis of species, and have proven to be an excellent indicator of the phylogenetic relationships among taxa (Cope 1896, Böhme 1988, Harvey et al. 2012). Harvey et al. (2012) concluded that the relationships among the genera of Tupinambinae, especially *Tupinambis* and *Salvator*, require further study, and that a more detailed analysis of hemipenial morphology, as well as muscles and osteology, may contribute to a more definitive understanding of the systematics of the group.

#### Measurements.

Based on eight specimens. Snout-vent length 135–260 mm (mean = 234.9 mm); body width 48.4–60.0 mm (mean = 54.0 mm), body height 30.5–44.6 mm (mean = 38.5 mm), head length 51.3–55.9 mm (mean = 53.6 mm), head width 33.9–45.0 mm (mean = 39.7 mm), head height 28.3–36.2 mm (mean = 31.2 mm). See Table 1 for a complete list of the measurements and scale counts recorded in the present study and those available in the literature (Manzani and Abe 1997, Colli et al. 1998, Silveira 2009).

#### Geographic distribution.

**Figure 2.**
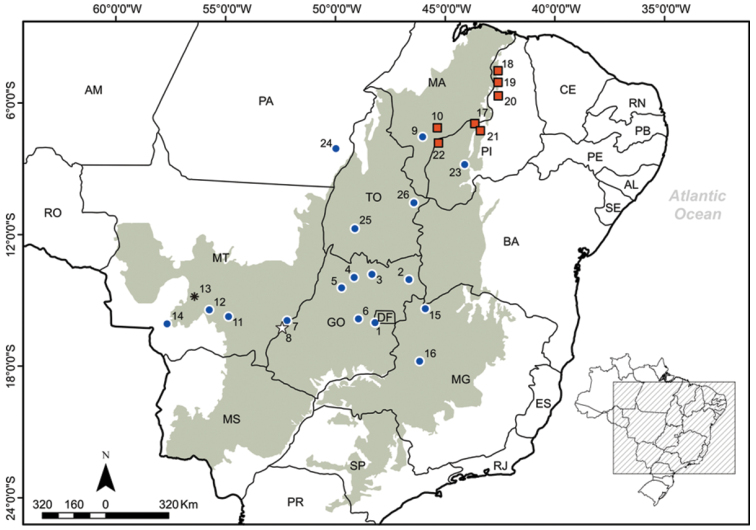
Known localities for *Tupinambis quadrilineatus* in Brazil. Distrito Federal (DF): Brasília, Gama (**1**). Goiás (GO): Iaciara (**2**) Minaçu (**3**) Mara Rosa (**4**) Santa Terezinha de Goiás (**5**) Pirenópolis (**6**) Aragarças (**7**) Baliza (**8**). Maranhão (MA): Balsas (**9**) São Raimundo das Mangabeiras (**10**). Mato Grosso (MT): Primavera do Leste (**11**) Chapada dos Guimarães (**12**) Rosário Oeste (**13**) Cáceres (**14**). Minas Gerais (MG): Chapada Gaúcha (**15**) João Pinheiro (**16**). Piauí (PI): Guadalupe (**17**) Lagoa Alegre (**18**) Altos (**19**) Monsenhor Gil (**20**) Amarante (**21**), Ribeiro Gonçalves (**22**) Uruçuí (**23**). Pará (PA): Santa Maria das Barreiras and Redenção (**24**). Tocantins (TO): Gurupi (**25**) Mateiros (**26**). The localities recorded in the present study are represented by red squares. The type-locality of *Tupinambis quadrilineatus* is shown as an asterisk, the type-locality of its junior-synonym (*Tupinambis cerradensis*) is shown as a star and remaining records from the literature are shown as blue circles (Manzani and Abe 1997; Colli et al. 1998; Guimarães et al. 2007; Silva Jr. et al. 2005; Vitt et al. 2005; Mesquita et al. 2006; Recoder and Nogueira 2007; Ferreira et al. 2009; Silveira 2009; Recoder et al. 2011; Dal Vechio et al. 2013). The Cerrado savanna biome is highlighted in gray.

The *Tupinambis* specimens available in Brazilian collections were examined together with the eight *Tupinambis quadrilineatus* specimens collected during the present study, in Maranhão and Piauí (Figure 2). The localities reported here represent the northernmost known records of *Tupinambis quadrilineatus*, and extend the known distribution of the species at least 500 km from the nearest locality, in Balsas, Maranhão (Barreto et al. 2007). This is the northernmost record of the occurrence of the species.

Five *Tupinambis quadrilineatus* specimens were examined in the collection of the Goeldi Museum. In 1993, specimen MPEG 16817 was collected in Balsas, Maranhão (reported by Barreto et al. 2007), and specimen MPEG 16845 was captured in the municipality of Lagoa Alegre, Piauí. In 2009, three specimens were collected during the Parnaiba Project in Ribeiro Gonçalves (MPEG 30139), and Uruçuí (MPEG 30141), in the state of Piauí, and São Raimundo das Mangabeiras (MPEG 30140), in Maranhão.

**Figure 3.**
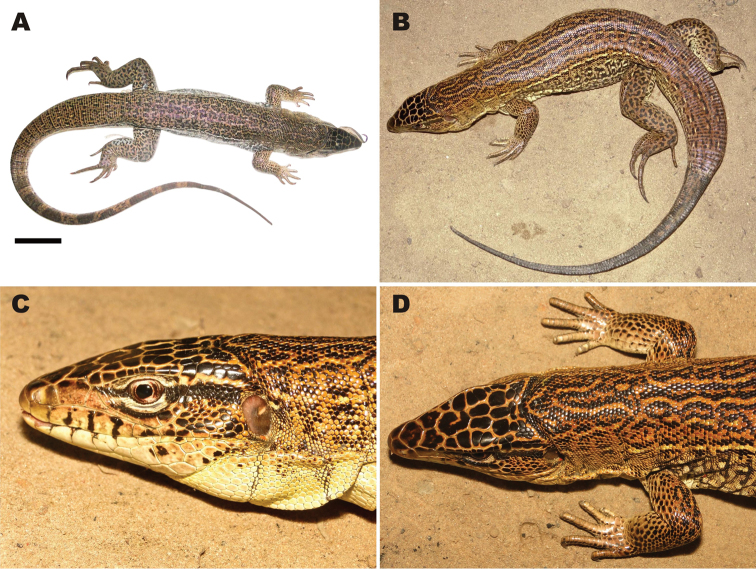
Adult male *Tupinambis quadrilineatus*. **A** specimen collected in the Palmares National Forest, Altos, Piauí (CHNUFPI 0036; Scale 5cm) **B** specimen collected with pit-fall traps at Guadalupe, Piauí (CHNUFPI 0038) **C** lateral view of the head and **D** dorsal view of the anterior region of the body (CHNUFPI 0036).

The herpetological collection of the Universidade Federal do Piauí provided specimens or records of *Tupinambis quadrilineatus* from a number of sites in Piauí. Specimen CHNUFPI 0036 (Figure 3A) was collected in 2010 in the Palmares National Forest (05°02'55"S, 42°35'59"W, SAD69), in the municipality of Altos. The vegetation of this area is semi-deciduous tropical forest typical of the Cerrado, an ecotonal region between Cerrado and Amazonia biomes, similar to that found in Lagoa Alegre. *Tupinambis quadrilineatus* occurs in syntopy with *Salvator merianae* in this area, as recorded at a number of other sites (Colli et al. 1998, Silveira 2009). Also in 2010, a roadkilled specimen of *Tupinambis quadrilineatus* (CHNUFPI 0037) was collected in the municipality of Monsenhor Gil (05°39'56"S, 42°35'28"W, SAD69). In May 2011, the third and final *Tupinambis quadrilineatus* specimen held in the collection (CHNUFPI 0038; Figure 3B–D) was collected in a pitfall trap installed in the vicinity of a small stream within an area dominated by Cerrado savanna (*sensu strictu*) in the municipality of Guadalupe (05°2'55"S, 42°35'59"W, SAD69). Two other specimens were observed in the municipality of Amarante (06°14'43"S, 42°46'46"W and 06°2'1"S, 43°3'40"W, SAD69) in 2009 and 2011, but specimens were not collected. In this area, the vegetation was dominated by secondary semi-deciduous tropical forest, mixed with patches of Cerrado *sensu strictu.*

These findings expand the geographic distribution of *Tupinambis quadrilineatus* is northwards, and encompass the the region between the states of Piauí and Maranhão, which is dominated by Cerrado *sensu strictu* and/or forested patches of the Cerrado–Amazon ecotone. In this region, *Tupinambis quadrilineatus* also occurs in syntopy with *Salvator merianae*, which was previously classified as a member of the genus *Tupinambis*.

## Supplementary Material

XML Treatment for Tupinambis quadrilineatus

## Appendix

### Material analyzed

**Table of localities.** *Tupinambis quadrilineatus*: **Maranhão**: MPEG 16817 (Rio Matões, right bank tributary of the Rio Balsas, Balsas), MPEG 30140 (São Raimundo das Mangabeiras), **Piauí:** CHNUFPI 0036 (Palmares National Forest, Altos), CHNUFPI 0037 (Monsenhor Gil), CHNUFPI 0038 (Fazenda São Pedro, Guadalupe), MPEG 16845 (Lagoa Alegre), MPEG 30139 (Ribeiro Gonçalves), MPEG 30141(Estação Ecológica de Uruçuí-Una, Uruçuí).

Collection	Collection number	Family	Genus	Species	Locality	Municipality	State	Latitude, Longitude	Coordinate origin
MPEG	MPEG 16817	Teiidae	*Tupinambis*	*quadrilineatus*	Near Matões River, tributary of the Balsas Rive	Balsas	Maranhão	7°31'S, 46°2'W	Google Earth
MPEG	MPEG 30140	Teiidae	*Tupinambis*	*quadrilineatus*		São Raimundo das Mangabeiras	Maranhão	7°1'S, 45°28'W	A. O. Maciel
CHNUFPI	CHNUFPI 0036	Teiidae	*Tupinambis*	*quadrilineatus*	Palmares National Forest	Altos	Piauí	05°02'55"S, 42°35'59"W	GPS
CHNUFPI	CHNUFPI 0037	Teiidae	*Tupinambis*	*quadrilineatus*	BR 343	Monsenhor Gil	Piauí	05°39'56"S, 42°35'28"W	GPS
CHNUFPI	CHNUFPI 0038	Teiidae	*Tupinambis*	*quadrilineatus*	São Pedro Farm	Guadalupe	Piauí	5°2'55"S, 42°35'59"W	GPS
MPEG	MPEG 16845	Teiidae	*Tupinambis*	*quadrilineatus*		Lagoa Alegre	Piauí	4°26'S, 42°35'W	Google Earth
MPEG	MPEG 30139	Teiidae	*Tupinambis*	*quadrilineatus*		Ribeiro Gonçalves	Piauí	7°33'S, 45°14'W	A. O. Maciel
MPEG	MPEG 30141	Teiidae	*Tupinambis*	*quadrilineatus*	Uruçuí-Una Ecological Station	Uruçuí	Piauí	7°14'S, 44°33'W	Google Earth
Not collected	Not collected	Teiidae	*Tupinambis*	*quadrilineatus*	BR 343	Amarante	Piauí	6°14'43.49"S, 42°46'46.02"W	GPS
